# Seasonal climate conditions impact the effectiveness of improving photosynthesis to increase soybean yield

**DOI:** 10.1016/j.fcr.2023.108907

**Published:** 2023-05-15

**Authors:** Yufeng He, Megan L. Matthews

**Affiliations:** a Carl R. Woese Institute for Genomic Biology, University of Illinois at Urbana-Champaign, IL 61801, USA; b Department of Civil and Environmental Engineering, University of Illinois at Urbana-Champaign, IL 61801, USA

**Keywords:** Photosynthetic assimilation, Field climate conditions, Soybean growth modeling, Soybean yield estimation, RuBP regeneration

## Abstract

**Context:**

Photosynthetic stimulations have shown promising outcomes in improving crop photosynthesis, including soybean. However, it is still unclear to what extent these changes can impact photosynthetic assimilation and yield under long-term field climate conditions.

**Objective:**

In this paper, we present a systematic evaluation of the response of canopy photosynthesis and yield to two critical parameters in leaf photosynthesis: the maximum carboxylation rate of ribulose-1,5-bisphosphate carboxylase/oxygenase (*V*_*cmax*_) and the maximum electron transport of the ribulose-1,5-bisphosphate regeneration rate (*J*_*max*_).

**Methods:**

Using the field-scale crop model Soybean-BioCro and ten years of observed climate data in Urbana, Illinois, U.S., we conducted sensitivity experiments to estimate the changes in canopy photosynthesis, leaf area index, and biomass due to the changes in *V*_*cmax*_ and *J*_*max*_.

**Results:**

The results show that 1) Both the canopy photosynthetic assimilation (*A*_*n*_) and pod biomass yields were more sensitive to the changes in *J*_*max*_, particularly at high atmospheric carbon-dioxide concentrations ([CO_2_]); 2) Higher [CO_2_] undermined the effectiveness of increasing the two parameters to improve *A*_*n*_ and yield; 3) Under the same [CO_2_], canopy light interception and canopy respiration were key factors that undermined improvements in *A*_*n*_ and yield; 4) A canopy with smaller leaf area index tended to have a higher yield improvement, and 5) Increases in assimilations and yields were highly dependent on growing-season climatic conditions. The solar radiation, temperature, and relative humidity were the main climate drivers that impacted the yield improvement, and they had opposite correlations with improved yield during the vegetative phase compared to the reproductive phase.

**Conclusions:**

In a world with elevated [CO_2_], genetic engineering crop photosynthesis should focus more on improving *J*_*max*_. Further, long-term climate conditions and seasonal variations must be considered to determine the improvements in soybean canopy photosynthesis and yield at the field scale.

**Implications:**

Quantifying the effectiveness of changing *V*_*cmax*_ and *J*_*max*_ helps understand their individual and combined contributions to potential improvements in assimilation and yield. This work provides a framework for evaluating how altering the photosynthetic rate parameters impacts soybean yield and assimilation under different seasonal climate scenarios at the field scale.

## 1 Introduction

Climate change and population growth are pressing problems and will continue to pose immediate risks to food security and increase demands on food production (Godfray and Garnett, 2014, Hasegawa et al., 2018). A central topic in plant science is to improve crop yields by increasing photosynthetic efficiency (Long et al., 2015; Ort et al., 2015; Raines, 2011; Simkin et al., 2019; Zhu et al., 2008). Transgenic engineering of the photosynthetic pathway has been shown to increase photosynthesis rates in a wide variety of crop types, such as tobacco (López-Calcagno et al., 2020), soybean (Hay et al., 2017), wheat (Parry et al., 2011), maize (Salesse-Smith et al., 2018) and rice (Ku et al., 2000).

Increasing carbon-dioxide concentration ([CO_2_]) in the atmosphere could lead to greater photosynthesis rates (Ainsworth and Rogers, 2007, Leakey et al., 2009b, Specht et al., 1999). In addition to [CO_2_], these rates are also highly dependent on other environmental factors, such as temperature and light. Model results also suggest alterations of the kinetic properties of Ribulose-1,5-bisphosphate carboxylase-oxygenase (Rubisco) and/or the regeneration of Ribulose-1,5-bisphosphate (RuBP) would increase photosynthetic assimilation rates under future climate conditions (Long et al., 2006). However, the extent to which these improvements would impact crop photosynthesis and yield under different field climate conditions is still unknown.

The correlation between assimilation and yield in annual crops is complex (Buttery et al., 1981, Evans and Fischer, 1999, Gifford and Evans, 1981, Specht et al., 1999). A recent study has shown that there is a large variability in the extent of these correlations with yield due to complex impacts across biological and temporal scales (Wu et al., 2023). For example, the effectiveness of photosynthetic enhancement could be undermined by increased photorespiration (Curtis et al., 1969) and nitrogen requirements (Sinclair and Wit, 1976, Yin et al., 2022). Further, it has been shown that a short-term, hourly or daily, gain in photosynthesis assimilation from increased [CO_2_] does not necessarily translate into a long-term yield gain (Sims et al., 1998). Particularly under constantly varying field conditions, the final yield is an integration of daily and hourly changes over the entire growing season. Therefore, determining the main contributor to photosynthesis at a seasonal scale is critical to evaluate the potential of photosynthetic improvements on yields. Using models can help us understand the overall sensitivities of assimilations and yields on changing climate conditions for a long climatically significant period, providing information for identifying new photosynthetic improvements.

In the steady-state biochemical model of photosynthesis, known as the Farquhar–von Caemmerer–Berry (FvCB) model (von Caemmerer, 2000), the net assimilation rate (*A*_*n*_) is limited by three processes: 1) *A*_*c*_, the rate of Rubisco carboxylation; 2) *A*_*j*_, the rate of RuBP regeneration, as determined by the electron transport fluxes; and 3) *A*_*p*_, the triose phosphate utilization (TPU) rate. The TPU rate has been found to rarely be the limiting factor at the current and the near-future atmospheric [CO_2_] levels due to its high internal [CO_2_] requirement (Kumarathunge et al., 2019, Sharkey, 2019). Most studies have focused on improving *A*_*c*_ and *A*_*j*_ to increase photosynthesis assimilation (López-Calcagno et al., 2020). Corresponding to *A*_*c*_ and *A*_*j*_ are two commonly used parameters: the maximum carboxylation rate of Rubisco (*V*_*cmax*_) and the maximum electron transport and RuBP regeneration rate (*J*_*max*_). These two high-level metrics are often used as measures of the photosynthetic efficiency of transgenic plants (López-Calcagno et al., 2020, Suzuki et al., 2009, Wu et al., 2019) or plants grown under different environments (Bernacchi et al., 2005, Galmés et al., 2015).

*V*_*cmax*_ and *J*_*max*_ have been increased by 10–30% by increasing the content and/or activity of certain photosynthetic enzymes in the Calvin–Benson cycle, resulting in an increase of 10–20% in *A*_*n*_ and leaf biomasses in tobacco (Lefebvre et al., 2005, López-Calcagno et al., 2020, Rosenthal et al., 2011, Simkin et al., 2015). Similar improvements have also been accomplished in transgenic soybeans with 4–14% increases in *A*_*n*_ and 4–8% increases in *V*_*cmax*_ and *J*_*max*_, which are expected to prevent a decrease in yield under the combined effects of future warming and elevated [CO_2_] (Köhler et al., 2016).

Most studies have focused on the sensitivities of environmental conditions, such as changing [CO_2_], temperature, and light (Cai et al., 2018), where *V*_*cmax*_ and *J*_*max*_ are treated as derived parameters from the *A-C*_*i*_ curve. However, this approach alone leaves a knowledge gap in systematically understanding the sensitivity of the two parameters under different environments. While *V*_*cmax*_ and *J*_*max*_ directly contribute to the magnitudes of *A*_*c*_ and *A*_*j*_, respectively, changing these parameters, either individually or together, alters how often each rate is limiting assimilation during a long-term simulation. Therefore, a comprehensive sensitivity study on the two parameters can determine the respective contributions of Rubisco and RuBP regeneration under a wide range of climate conditions, which can be used to support effective lab testing. This information may further help identify strategies for improving photosynthesis best suited for current and future climate conditions.

When designing strategies for increasing photosynthetic assimilation and yield it is also essential to consider canopy gradients and micro-climates. Not only can climate alter crop development progress (He et al., 2020), but the impacts of the same climate condition during different developmental phases will have varying effects on crop yield (Wu et al., 2023). For example, an optimum light and temperature condition may differ in determining the final yield for the reproductive and vegetative phases. Many real-world conditions that can be simulated by advanced crop growth models are difficult, if not impossible, to replicate in the lab. These conditions may include light attenuation due to leaf canopy shading effects, constant radiation changes due to cloud cover, and extreme weather conditions in temperature and precipitation. Using real climate field data instead of controlled lab data, we can have a more realistic response of photosynthesis and yield and explore the variabilities in climate driving forcing and predicted quantities.

In this paper, we use a crop growth model, Soybean-BioCro (Matthews et al., 2022), to simulate soybean growth using observed climate data and quantify the impacts of changing *V*_*cmax*_ and *J*_*max*_ on *A*_*n*_ and yields. We further evaluate how climate conditions impact the effectiveness of increasing *V*_*cmax*_ and *J*_*max*_ at a seasonal scale. Our specific objectives are to 1) estimate the changes in *A*_*n*_ and yields with a range of changes in *V*_*cmax*_ and *J*_*max*_ under current and future [CO_2_] conditions; 2) examine the causes of reduced returns in *A*_*n*_ and yields for a 20% increase in *V*_*cmax*_ and *J*_*max*_; and 3) quantify the contribution of each climate driver (including solar radiation, air temperature, precipitation, relative humidity and wind speed) to the yield gain for the vegetative and reproductive phases respectively.

## 2 Material and methods

### 2.1 Model description

All of the field-scale soybean simulations were implemented using Soybean-BioCro, a crop growth model that is part of the BioCro model framework (Lochocki et al., 2022, Matthews et al., 2022) . The model calibration and validation for Soybean-BioCro were conducted against four years (i.e., 2002, 2004, 2005, and 2006) of observed climate data, as well as soybean biomass measurements (Pioneer 93B15) collected at the SoyFACE facility (40.04°N, 88.23°W) at the University of Illinois at Urbana-Champaign (Matthews et al., 2022, Morgan et al., 2005). Soybean-BioCro incorporates the Farquhar–von Caemmerer–Berry (FvCB) model and Ball-Berry stomatal conductance model to simulate a 10-layer canopy photosynthesis that contains sunlit and shaded leaves (Fig. S1). It then calculates photothermal development rate and uses logistic functions to partition assimilated carbon into biomass. The growth and senescence of leaf, stem, grain, and root biomasses are estimated at an hourly time step and integrated throughout the growing season (Matthews et al., 2022).

### 2.2 Definition of main quantities

We define the following metric for representing the relative change of variables,

(1) $$\mathrm{Relative}\;\;\mathrm{change}=\frac{\mathrm{VxJx}-\mathrm{CTL}}{\left|\mathrm{CTL}\right|}*100\%$$

Where CTL is the model simulation with the default values of *V_cmax_* and *J_max_*. VxJx represents the simulation experiments with changed values in the two parameters, where *x* represents the amount of change in percentage.

The photosynthetic water use efficiency (WUE) at the canopy level (Medrano et al., 2015) is calculated as,

(2) $$\mathrm{WUE}=\frac{A_{n}}{E}(\mu \mathrm{mol}\;\;{\mathrm{mmol}}^{-1})$$

Where *A*_*n*_ is the canopy-level net assimilation rate, and *E* is the canopy-level evapotranspiration rate. The leaf level evapotranspiration is estimated by the Penman-Monteith evapotranspiration model, which is then integrated over the canopy layers to obtain *E* (Fig. S1).

### 2.3 Data and experiment design

Ten years of weather data from 2006 to 2015 in Bondville, Illinois, were used for the model simulations. The driving climate variables include air temperature, precipitation, solar radiation, relative humidity, and wind speed. All observed climate data were obtained from the Surface Radiation Budget Network (https://gml.noaa.gov/grad/surfrad/) except for the precipitation. The precipitation data were received from the Illinois Climate Network (https://www.isws.illinois.edu/dat/).

Two key parameters in the FvCB model, the maximum carboxylation rate of Rubisco (*V*_*cmax*_) and the maximum electron transport and RuBP regeneration rate (*J*_*max*_), were investigated in a series of model sensitivity experiments. For the control experiments (CTL), the default values of the two parameters are, *V*_*cmax*_= 110 and *J*_*max*_ = 195 *μmol m*^*−2*^ *s*^*−1*^ (Matthews et al., 2022). For the sensitivity experiments (VxJx), we scaled *V*_*cmax*_ and *J*_*max*_ by a range of values from − 50% to + 50% at a 5% step size. Four [CO_2_] levels were simulated: 400, 600, 800, and 1000 ppm, where 400 ppm is approximately the current level of the atmospheric [CO_2_]. The 600 and 1000 ppm values are in the range of the predicted [CO_2_] in an extreme scenario for 2050 and 2100, respectively (Joos et al., 2001). The abbreviation V20J20 was used to represent the experiments with a 20% increase in both *V*_*cmax*_ and *J*_*max*_.

We then estimated the absolute and relative changes (Eq. 1) in six selected variables of interest: the harvestable biomasses of pod and shoot (sum of pod, leaf, and stem), seasonal averages of daily maximum and mean of *A*_*n*_, maximum leaf area index (LAI) during the growing season and average of daily mean WUE. The relative changes represent the effectiveness of changing *V*_*cmax*_ and *J*_*max*_ at corresponding climate and [CO_2_] conditions. Unless otherwise specified, *A*_*n*_ refers to the canopy-level *A*_*n*_.

### 2.4 Gradient descent

To find the steepest path on the heatmap of the relative change against *V*_*cmax*_ and *J*_*max*_, we used a simple gradient descent algorithm as follows,

(3) $$X_{n+1}=X_{n}-\alpha \nabla f\left(X_{n}\right)$$

Where *X*_*n*_ is a two-dimensional coordinate of *V*_*cmax*_ and *J*_*max*_ at the *n*_*th*_ step. α is the step size of path searching (α=0.001 was used in the simulations). The gradient of the function *f* can be calculated as,

(4) ∇fX=∂f∂x∂f∂y

Where *x* and *y* represent the *V*_*cmax*_ and *J*_*max*_, respectively. To estimate the derivatives of discrete functions, we used the following central difference approximation method,

(5) $$\frac{\partial f}{\partial x}=\underset{h\to 0}{\lim}\frac{f\left(x+h,y\right)-f\left(x-h,y\right)}{2h}$$

(6) $$\frac{\partial f}{\partial y}=\underset{h\to 0}{\lim}\frac{f\left(x,y+h\right)-f(x,y-h)}{2h}$$

Where *h= 0.01*, and *f(x,y)* is estimated using a non-linear spline interpolation from the Akima package in R (Akima & Gebhardt, 2022).

### 2.5 Bootstrap of climate data and partial rank correlation

To better represent the day-to-day variability of the 10-year observed climate data, we used a bootstrap method to create 1000 scenarios of annual climate data based on a re-sampling process for all days of the year (DOY). For each bootstrapped climate scenario, all of the climate drivers were randomly sampled on a daily basis from one of the 10 years of weather data. For a given DOY, all 24 hours for all of the climate variables were sampled from the same year. This sampling strategy preserves the diurnal relationships and any relationships between the climate drivers (e.g., sunlight and temperature, precipitation and humidity). The bootstrapped climate scenarios were statistically generated to better represent the 10-year variability, and do not represent future climate conditions.

To evaluate the relationships between pod biomass gains and climate conditions at the vegetative and reproductive phases, we used partial rank correlation to represent each climate driver’s contribution to the biomass gain. The partial rank correlation coefficients (PRCC) were calculated using the epiR package in R (Stevenson et al., 2022).

## 3 Results

### 3.1 Sensitivity of assimilation, LAI, and yield to the changes in *V*_*cmax*_ and *J*_*max*_

**Fig. 1:**
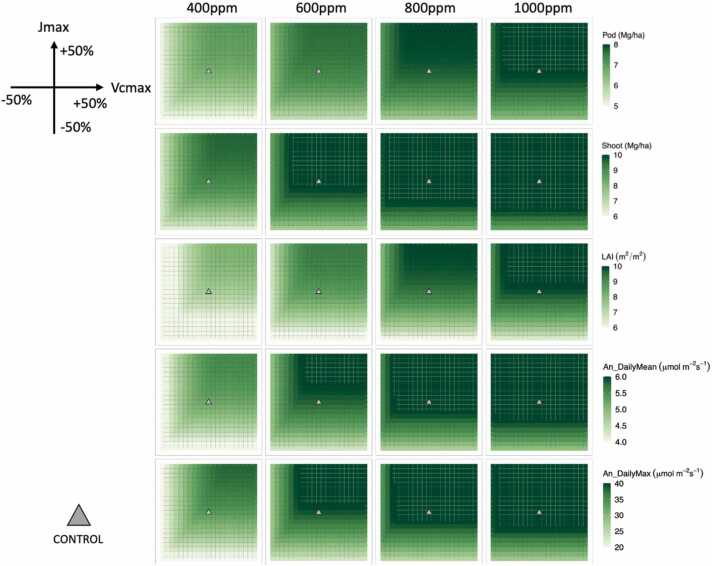
Heatmaps of model estimations of the five variables at four [CO_2_] levels (400, 600, 800, and 1000 ppm) in responses to the changes in *V*_*cmax*_ and *J*_*max*_. The variables include annual pod and shoot biomass, maximum LAI, daily mean, and maximum net assimilation rate (An). The control experiment (grey triangle) has the default values of *V*_*cmax*_ (=110) and *J*_*max*_ (=195). The values shown were calculated by the ten-year averages from 2006 to 2015.

Biomasses, LAI, and *A*_*n*_ were all impacted by the changes in *V*_*cmax*_ and *J*_*max*_. Larger values were predicted for all variables as the atmospheric [CO_2_] increased (Fig. 1). At 400 ppm, the pod and shoot biomasses ranged from 4.9–7.0 and 6.3–9.6 Mg/ha, respectively, compared with their controls of 6.6 and 9.1 Mg/ha. At 800 ppm, these ranges shifted to 6.1–8.0 and 8.1–11.2 Mg/ha with the controls of 7.7 and 10.8 Mg/ha. Similar trends were observed at 400 and 800 ppm for: LAI with ranges of 4.3–8.1 and 5.4–9.9 m^2^/m^2^ and the respective controls of 7.3 and 9.3 m^2^/m^2^^,^ daily mean *A*_*n*_ with ranges of 3.6–5.5 and 4.7–6.4 *μmol m*^*−2*^ *s*^*−1*^ and the controls of 5.2 and 6.2 *μmol m*^*−2*^ *s*^*−1*^^*,*^ and daily maximum *A*_*n*_ with ranges of 18.4–37.9 and 26.1–46.2 *μmol m*^*−2*^ *s*^*−1*^ and the controls of 33.4 and 42.3 *μmol m*^*−2*^ *s*^*−1*^.

**Fig. 2:**
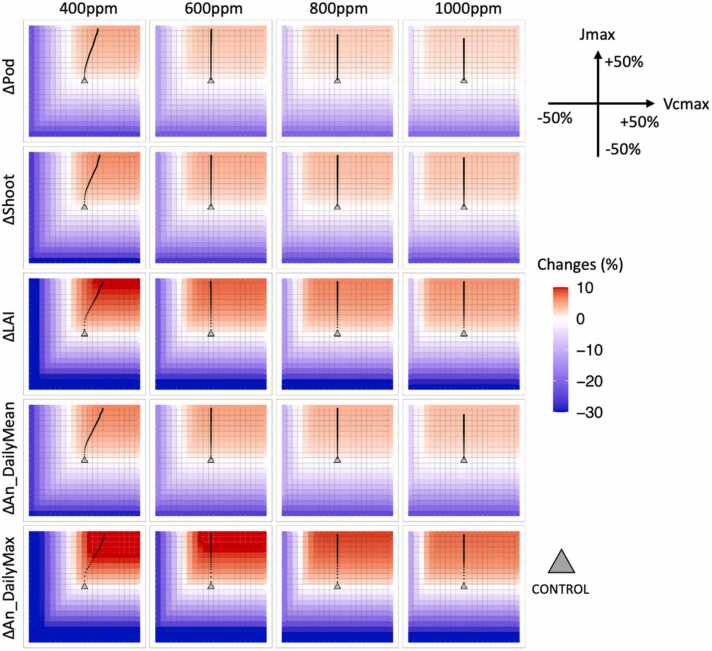
Heatmaps of the changes (Δ) in five variables at four [CO_2_] levels in responses to the changes in *V*_*cmax*_ and *J*_*max*_. The variables include annual pod and shoot biomass, maximum LAI, daily mean, and maximum net assimilation rate (An). The colors represent the level of changes (in %) of each sensitivity experiment regarding the control experiment (grey triangle). The values were calculated by the ten-year averages. The black dotted lines represent the fastest-changing paths with the largest gradients.

Although higher [CO_2_] increased assimilation and yield in general, the increases were seen in both the sensitivity experiments (VxJx) and the corresponding controls (CTL). To better understand the effectiveness of changing *V*_*cmax*_ and *J*_*max*_ we quantified the relative difference between VxJx and CTL at all [CO_2_] levels (Fig. 2). Varying *V*_*cmax*_ and *J*_*max*_ rates had a more significant impact on assimilation and yield under lower levels of atmospheric [CO_2_] (Fig. 2). When compared with their controls, increasing both *V*_*cmax*_ and *J*_*max*_ at 400 ppm resulted in increases of up to 18% in the maximum *A*_*n*_, 13% in the LAI and 8% in both the shoot and pod biomasses (Fig. 2). Decreasing *V*_*cmax*_ and *J*_*max*_ resulted in losses of up to 46% in the maximum *A*_*n*_, 43% in the LAI, 33% in the shoot and 29% in the pod (Fig. 2). This trend was also observed at higher [CO_2_], but with a decreasing range of impacts as [CO_2_] increased, such as the pod biomass which was only increased by 4.2% at 800 ppm versus 8% at 400 ppm (Fig. 2).

**Fig. 3:**
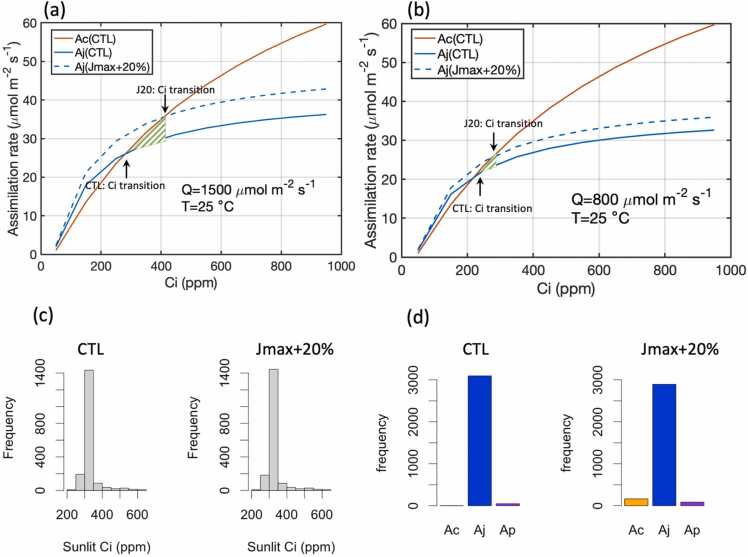
(a) A-Ci curves of the *A*_*c*_ and *A*_*j*_ for the control case and the *A*_*j*_ with a 20% increase in *J*_*max*_ at a light intensity (Q) of 1500 μmol m^−2^ s^−1^. (b) Same as (a) but with a light intensity (Q) of 800 μmol m^−2^ s^−1^. (c) Frequency of the three limiting factors for the sunlit top layer in the Soybean-BioCro hourly simulations. (d) Frequency of the *C_i_* levels for the sunlit top layer. This example used the data for the growing season of 2006 at 400 ppm [CO_2_]. The A-Ci curves were obtained using the leaf-level Farquhar model with the same parameters as the full FvCB model in the Soybean-BioCro.

In all simulated scenarios, the variables of interest were more sensitive to changes in *J*_*max*_ than *V*_*cmax*_. Starting from the control experiments (Fig. 2, gray triangles), the path with the largest ascending gradient was mainly determined by increasing *J*_*max*_, particularly at [CO_2_] above 400 ppm, where increasing *V*_*cmax*_ had little to no impact as indicated by the gradient paths (Fig. 2, black dotted lines). The model was more sensitive to changes in *J*_*max*_ as the assimilation rate was almost entirely limited by *A*_*j*_*,* the rate of RuBP regeneration (Fig. 3b). To achieve maximal gains at 400 ppm, however, increasing *V*_*cmax*_ was still necessary to maximize the yield and assimilation as increasing *J*_*max*_ increased the transition point of the intracellular carbon, *C*_*i*_, where the limiting rate switched between *A*_*c*_ and *A*_*j*_ from ∼280 ppm to ∼410 ppm at high light (Fig. 3a). At a lower light condition of 800 *μmol m*^*−2*^ *s*^*−1*^, the transition point of *C*_*i*_ was reduced to ∼220 ppm and ∼280 ppm (Fig. 3b). At 400 ppm of [CO_2_], the hourly *C*_*i*_ showed few occurrences below 250 ppm and a small amount between 250 and 300 ppm (Fig. 3c). Consequently, more instances of *A*_*c*_ occurred as the limiting rate when the transition point of *C*_*i*_ increased (Fig. 3d). Thus, at current levels of atmospheric [CO_2_], increasing both *J*_*max*_ and *V*_*cmax*_ were required to maximize assimilation, but at a higher [CO_2_] the system became much less likely to be limited by *A_c_*, therefore neglecting the contributions from increased *V*_*cmax*_. In fact, at a higher [CO_2_], *V*_*cmax*_ can even be decreased to 85% of its control without any negative impact on assimilation or biomass if *J*_*max*_ were unchanged or increased (Fig. 2).

**Fig. 4:**
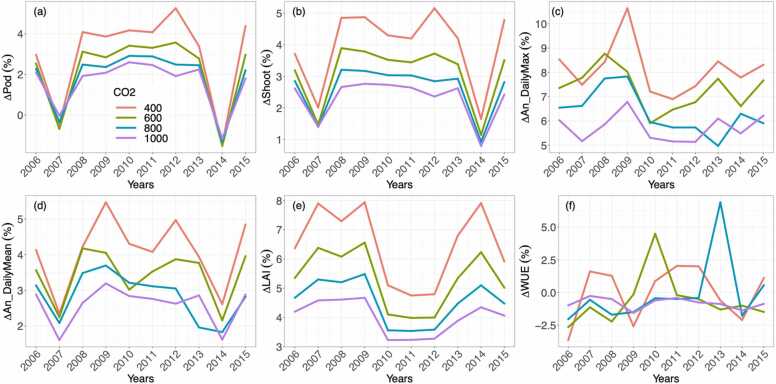
Impacts of increasing *V*_*cmax*_ and *J*_*max*_ by 20% (V20J20) at four [CO_2_] levels on the relative changes (%) of (a) Pod, (b) Shoot, (c) average of daily maximum *A*_*n*_ throughout the growing season, (d) average of daily mean *A*_*n*_, (e) max LAI during the growing season and (f) average of WUE. Δ = V20J20 – CTL.

The results from the sensitivity analysis (Fig. 1, Fig. 2) represent each variable's interannual averages over the ten years. However, interannual variations were significant for all variables within those ten years. For example, increasing both *V*_*cmax*_ and *J*_*max*_ by 20% resulted in larger relative gains in some of the simulated years than in others (Fig. 4). At 400 ppm, the daily maximum *A*_*n*_ ranged from a 6.9%−10.6% increase (Fig. 4c), followed by the peak LAI with gains of 4.7%−7.9% (Fig. 4e), the daily mean An ranged from a 2.3%−5.5% increase (Fig. 4d), the shoot biomass ranged from a 1.6%−5.1% increase (Fig. 4b), and the pod biomass ranged from a -1.4%−5.3% change (Fig. 4a). In two of the ten years, the pod biomass was lower in the V20J20 scenario than the CTL. In 2007, there was a 0.5% loss and in 2014 there was a 1.4% loss in pod biomass. In the other eight years, the pod biomass increased by 3%−5.3% (Fig. 4a). Overall, the relative gains for each variable became consistently smaller as [CO_2_] increased, but similar interannual patterns were predicted. The changes in WUE showed little similarity among the [CO_2_] scenarios. Although there was a significant interannual variation over the simulated period, the 10-year average of the relative changes was minimal compared with the other variables (Fig. 4f).

### 3.2 Key factors that limit the effectiveness of increasing *V*_*cmax*_ and *J*_*max*_

**Fig. 5:**
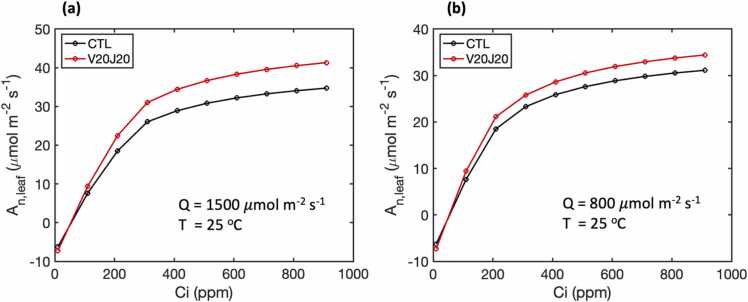
Relationships between net assimilation rate (*A*_*n*_) and intercellular CO_2_ concentration (*C*_*i*_) at the leaf level under two light conditions, (a) Q = 1500 *μmol m*^*−2*^*s*^*−1*^ and (b) Q = 800 *μmol m*^*−2*^*s*^*−1*^. The sensitivity experiment (V20J20) has a 20% increase in both *V*_*cmax*_ and *J*_*max*_. The A_n_-C_i_ curves are obtained using the leaf-level Farquhar model with the same parameters as the full FvCB model used in this study.

Although the daily maximum *A*_*n*_ increased more than the other variables, it was still limited to a 10% increase when *V*_*cmax*_ and *J*_*max*_ were both increased by 20% at 400 ppm (Fig. 4). The improvements in the daily maximum *A*_*n*_ were even smaller at higher [CO_2_]. This reduced gain in assimilation and yield was largely determined by the amount of light received by the canopy. At a high light condition of 1500 *μmol m*^*−2*^ *s*^*−1*^, increasing *V*_*cmax*_ and *J*_*max*_ by 20% resulted in an increase in the leaf-level *A*_*n*_ by approximately 18% when *C*_*i*_ was between 400 and 600 ppm (Fig. 5a). While at a low light condition of 800 *μmol m*^*−2*^ *s*^*−1*^, the leaf-level *A*_*n*_ was only increased by 8% (Fig. 5b).

The leaf-level photorespiration was also greater with the increased *V*_*cmax*_ and *J*_*max*_ (Fig. S2). However, the increases in photorespiration were rather small when compared with the increases in carboxylation under both high and low light conditions. At a *C*_*i*_ of 400 ppm, photorespiration was only increased by 10% of the increase seen in the carboxylation rate (Table S1). At a higher *C*_*i*_ of 600 ppm, the increase in photorespiration was only 7% of the increase in carboxylation (Table S1).

**Fig. 6:**
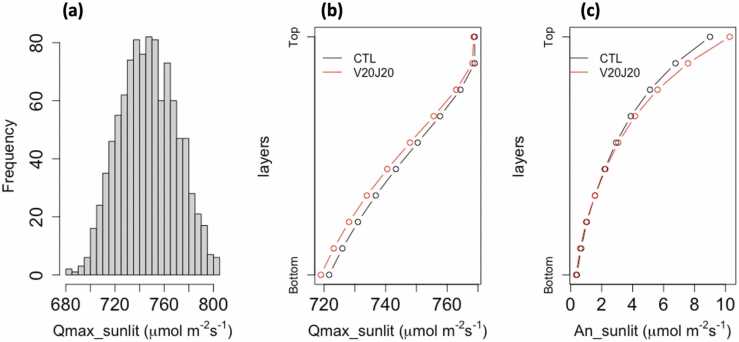
(a) Distribution of Qmax_sunlit for all layers and 1000 bootstrap samples for the CTL scenarios. (b) 10-layer profile of daily max radiation on the sunlit canopy (Qmax_sunlit) at [CO_2_] of 400 ppm. (c) 10-layer profile of the maximum *An* of the sunlit canopy (An_sunlit). Both Qmax_sunlit and An_sunlit represent quantities per leaf area.

In the Soybean-BioCro simulations, the incoming light was mainly received by the sunlit part of the canopy. Throughout the growing season, the daily maximum solar radiation received by the sunlit canopy was about 700–800 *μmol m*^*−2*^ *s*^*−1*^ (Fig. 6a) due to the light interception calculated using an average leaf orientation of the canopy. Slightly lower sunlit radiation was estimated for the scenarios with increased *V*_*cmax*_ and *J*_*max*_ (V20J20) among all canopy layers, mostly distinctive at the bottom layers (Fig. 6b). This was due to a larger LAI simulated for the V20J20 scenarios, which caused more of the lower layers to be shaded. Consequently, there was a diminished effect on the daily maximum *A*_*n*_ at the bottom layers while there was a more prominent effect at the top layers (Fig. 6c). The combined effect from all layers determined the amount that *A*_*n*_ was increased.

The canopy dark respiration (*R*_*d*_) was also increased with increased *V*_*cmax*_ and *J*_*max*_, which led to further losses on the *A*_*n*_. However, when compared with gross assimilation, *R*_*d*_ only contributed to a small percentage in the daily maximum *A*_*n*_ (Fig. S3a & c) when there was high light in a day. Therefore, the increases in the daily maximum *A*_*n*_ (∼8%) were close to those estimated at the leaf-level at 400 ppm (Fig. 5b). The contribution of *R*_*d*_ became particularly significant when considering the daily mean *A*_*n*_ (Fig. S3b & d), where changes in *R*_*d*_ can surpass changes in gross assimilation under low light and dark conditions. Therefore, the increases in *A*_*n*_ were further undermined (Fig. 4d) in its daily mean when compared with its daily maximum (Fig. 4 c).

### 3.3 Main climate drivers that determine the yield improvement

**Fig. 7:**
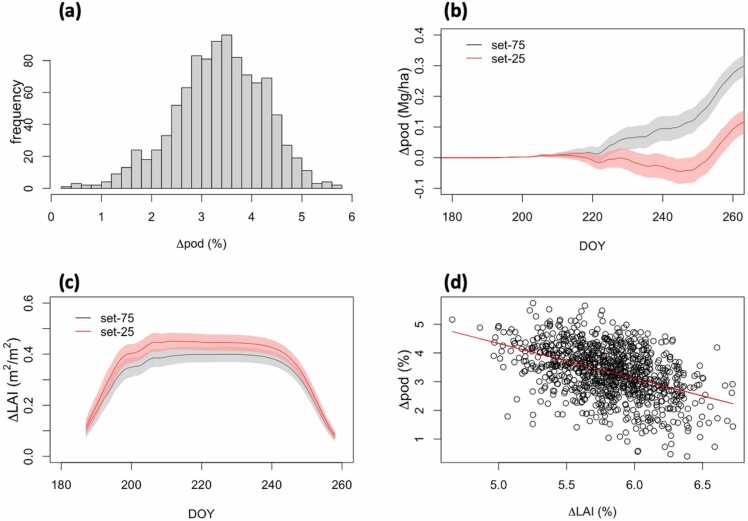
Changes in pod biomass and LAI due to an increase of *V*_*cmax*_ and *J*_*max*_ by 20% at [CO_2_] of 400 ppm driven by bootstrap climate samples. (a) Frequency distribution of pod gains (Δpod, %) for the 1000 bootstrap samples. (b) Time series of the absolute difference of pod biomass (Δpod, Mg/ha), separated by lower (set-25; red line) and upper (set-75; black line) quantiles. Shaded bands represent one standard deviation for each set. (c) Same as (b), but for the difference of maximum LAI (ΔLAI). (d) The relationship between ΔLAI (%) and Δpod (%) with a linear regression line (red).

One thousand bootstrapped scenarios were generated from the ten-year observed climate data to better represent the climatic variability. Increases in pod biomass (Δpod) varied from 0.4% to 5.7% among the bootstrapped samples when *V*_*cmax*_ and *J*_*max*_ were increased by 20% at the [CO_2_] of 400 ppm (Fig. 7a). The lower quantile consisting of 250 samples (set-25) predicted 2.2% ± 0.5% in Δpod, and the upper quantile (set-75) predicted 4.4% ± 0.4% in Δpod. The Δpod time series for the two sets started to diverge from the beginning of the predicted reproductive phase on about DOY 210 until the end of the growing season (Fig. 7b). A significant difference in the maximum LAI changes (ΔLAI) was found between the two sets (Fig. 7c), where set-25 predicted consistently higher ΔLAI than set-75, which was inversely correlated with their Δpod predictions. This was further confirmed by a significant negative correlation (*r* = −0.41, *p* < 0.001) between ΔLAI and Δpod for all bootstrap samples (Fig. 7d).

**Fig. 8:**
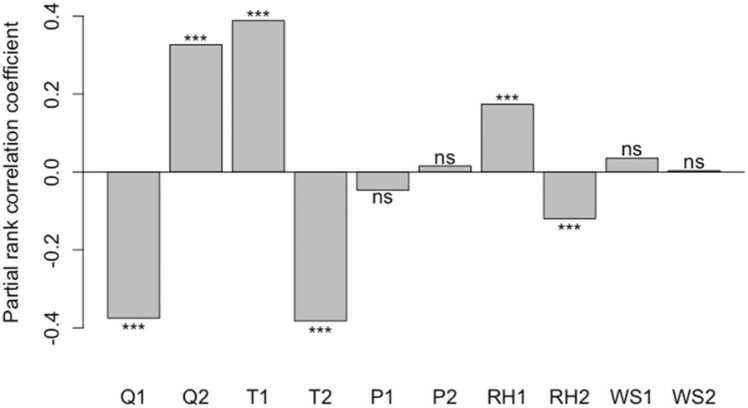
Partial rank correlation coefficients between Δpod and climate variables are separated into the vegetative phase (indicated by 1) and reproductive phase (indicated by 2). The five climate variables are solar radiation (Q), air temperature (T), precipitation (P), relative humidity (RH), and wind speed (WS). The climate variables are the 1000 bootstrap samples from the 10-year observed data in Illinois. The three-asterisk symbols represent a significance level of the coefficients with p-values less than 0.001. The ns symbol stands for not significant.

The large variation in the predicted Δpod originated from different growing-season climate conditions that were generated using the bootstrap sampling of the 10-year period previously examined. Two growth phases of soybean are the vegetative and reproductive phases. Climate conditions during each of these phases contributed to Δpod differently. Among the five climate variables, solar radiation (Q), air temperature (T), and relative humidity (RH) showed significant partial rank correlations with Δpod at both growth phases (Fig. 8). During the vegetative phase, Q was negatively correlated with Δpod (PRCC=−0.37, p < 0.001), while T and RH were positively correlated with Δpod (PRCC=0.39 & 0.17, p < 0.001). In comparison, these correlations were reversed during the reproductive phase with similar correlation levels (Fig. 8). The opposing correlations between the two phases can be explained by examining the relationship between the climate drivers and Δ*A*_*n*_/ΔLAI. In both phases, T was negatively correlated with Δ*A*_*n*_ and Q was positively correlated with Δ*A*_*n*_ (Fig. S4). Since a higher *A*_*n*_ during the vegetative phase led to a higher LAI and given that ΔLAI correlated negatively with Δpod (Fig. 7d), Δ*A*_*n*_ was negatively correlated with Δpod (Fig. S5a). A higher *A*_*n*_ during the reproductive phase, however, did not impact LAI and contributed directly to pod biomasses (Fig. S5b). Correlations between Δpod and the other two climate variables, precipitation and wind speed, were not statistically significant under the climate conditions at the study site (Fig. 8). The model simulations did not show significant water stress, so the results and their interpretations are limited to water non-limiting conditions.

## 4 Discussion

### 4.1 *Increasing J*_*max*_ contributes more to the improvements of assimilation and yield

At the current [CO_2_] of about 400 ppm, increasing the rate of Rubisco carboxylation has little impact, and thus there is a low potential for improving crop photosynthesis through increasing this rate in soybean (Ainsworth and Rogers, 2007, Leakey et al., 2009a, Wise et al., 2004). This conclusion is consistent with our model prediction that a more significant contribution was found in *J*_*max*_ than *V*_*cmax*_ (Fig. 2). At a high [CO_2_] condition, assimilation is mainly limited by the capacity for RuBP regeneration (Long et al., 2004), same as our model representation of *A*_*j*_ being the main limiting factor under increased atmospheric [CO_2_] (Fig. 3).

Previous modeling studies have demonstrated a possible nitrogen overinvestment in Rubisco in existing annual crops (Wu et al., 2019, Yin et al., 2022). At the higher [CO_2_] level predicted for the future, there is an even smaller, and sometimes negligible, impact from increasing *V*_*cmax*_, implying that Rubisco activity could be decreased without losing photosynthetic benefits from increased *J*_*max*_ (Fig. 2). Therefore, a potential pathway to further boost assimilation could focus on reallocating the resources like nitrogen from Rubisco to other parts of the plant.

### 4.2 A higher [CO_2_] lowers the effectiveness of increasing *V*_*cmax*_ and *J*_*max*_

Higher atmospheric [CO_2_] improved yields in both the CTL and VxJx simulations (Fig. 1), but the effectiveness of increasing *V*_*cmax*_ and *J*_*max*_ was reduced as [CO_2_] increased (Figs. 2 & 4). A previous experimental study reported that transgenic soybeans showed larger *V*_*cmax*_ and *J*_*max*_ than the wildtypes under both ambient and elevated [CO_2_] (Hay et al., 2017). The effectiveness of changing *V*_*cmax*_ and *J*_*max*_ can be viewed as the change in *A*_*n*_ per unit change of *V*_*cmax*_ or *J*_*max*_. Following this definition, it is evident that increasing *V*_*cmax*_ or *J*_*max*_ was more effective in enhancing *A*_*n*_ at a lower [CO_2_] (Hay et al., 2017). However, an increase in yield was still predicted under elevated [CO_2_] with a higher *V*_*cmax*_ and *J*_*max*_ compared to the control (Fig. 1).

### 4.3 Why were increases in *A*_*n*_ and yield much lower than in *V*_*cmax*_ and *J*_*max*_?

Significant reductions in the returns in *A*_*n*_ and yield have been found in both C3 and C4 crops with boosted photosynthesis (Sinclair et al., 2004, Wu et al., 2018). Similarly, we estimated that a 20% increase in *V*_*cmax*_ and *J*_*max*_ led to less than a 10% gain in the maximum *A*_*n*_ and even lower gains in the mean *A*_*n*_ and pod biomasses (Fig. 4). The reduced gain in the maximum *A*_*n*_ was primarily due to the light interception at the canopy level. The percentage gain in *A*_*n*_ can approach 20% under high light, but is only around half of that under low light (Fig. 5). Canopy light use efficiency is a key factor that impacts photosynthesis and yield (Koester et al., 2014, Yin et al., 2022), therefore identifying optimal canopy structures could further improve the effectiveness of stimulating photosynthesis.

*R*_*d*_ was another key factor that undermined the gain in *A*_*n*_, particularly in the daily mean *A*_*n*_ due to diurnal variations of the increases in both gross assimilation and *R*_*d*_ (Fig. S3). It is important to consider both day and night hours to calculate the daily mean since the yield is continuously accumulated. This explains why increases in the daily mean *A*_*n*_ were much closer to and better correlated with gains in the yield than that in the daily maximum *A*_*n*_ (Fig. 4).

The increases in pod biomass were even less than that in the daily mean *A*_*n*_ due to additional losses from the development and carbon allocation processes, which can further undermine the effectiveness of increased *V*_*cmax*_ and *J*_*max*_. The biomass partitioning varies significantly at different crop development stages (Matthews et al., 2022), causing a heterogeneous transfer of assimilated carbon into the final yields. Sensitivity analyses, as shown in this study, use coupled canopy photosynthesis and carbon allocation models and can reveal complex input-output information during the entire crop development period, which is helpful for decision-making toward an effective photosynthetic improvement to increase yield.

### 4.4 Canopy with smaller LAI can be more effective

Most of the light absorption occurs at the top of the canopy, with photosynthetic assimilation decreasing with depth (Amthor, 1994). Using a multiple-layer canopy model, we were able to capture an important feature that a soybean canopy with smaller LAI was generally more effective in gaining biomass for increased *V*_*cmax*_ and *J*_*max*_ (Fig. 7). This is mainly due to the changed light use among the vertical canopy profile, where a smaller canopy has decreased shading from top layers, increasing the light received by the lower leaves and thus producing a higher yield overall (Fig. 6). Since leaf growth mainly occurs during the vegetative phase, lower radiation and/or higher temperature during this period would lead to a smaller LAI and thus a higher yield (Fig. 8, S4 & S5). Previous experimental studies also found similar results, and an optimal LAI may exist for obtaining the highest efficiency in canopy photosynthesis, which does vary with crop types and environmental conditions (Srinivasan et al., 2017, Tagliapietra et al., 2018).

### 4.5 On WUE

We found no significant changes in WUE for the simulated 10-year period when considering only the changes of *V*_*cmax*_ and *J*_*max*_ (Fig. 4f). This is because the canopy assimilation and transpiration can change simultaneously. Two studies on the photosynthetic stimulation of tobacco have demonstrated opposite results. (López-Calcagno et al., 2020) found increased WUE with increased *V*_*cmax*_ and *J*_*max*_, while (Simkin et al., 2015) reported that these increases in *A*_*n*_ were accompanied by increased stomatal conductance and decreased WUE. Whether changing the two parameters would impact the WUE seems inconclusive. While yield is the primary focus for photosynthetic improvements, evaluating WUE is crucial to understanding regional water sustainability, breeding resilience under extreme climate conditions like drought, and designing optimal cropping systems with soybean (Baath et al., 2021).

## 5 Conclusions

Despite many efforts spent on improving plant photosynthetic assimilation and yield by increasing *V*_*cmax*_ and *J*_*max*_, how effective such an approach is under varying environmental conditions remained uncertain. In this paper, we used a semi-mechanistic crop growth model, for the first time, to systematically evaluate the effectiveness of increasing *V*_*cmax*_ and *J*_*max*_ on soybean growth using real field climate data and its statistical synthetics. The detailed results showed how changes in *A*_*n*_ and biomasses respond to the changes in *V*_*cmax*_ and *J*_*max*_ under different [CO_2_] and field climatic conditions. This work provides a framework for evaluating the effectiveness of increasing or decreasing *V*_*cmax*_ and *J*_*max*_ on assimilation and yield. While it is crucial to explore the potential of boosting leaf photosynthesis at the plant level, to achieve better and potentially maximum effectiveness of these improvements, we should also consider designing better canopy structures and incorporate the impacts of seasonal climate variability in the region of interest.

## Declaration of Competing Interest

The authors declare that they have no known competing financial interests or personal relationships that could have appeared to influence the work reported in this paper.

## Data Availability

All codes and data used in this study are free to be downloaded at the GitHub repository (https://github.com/cropsinsilico/Soybean-Sensitivity).
