# Supplementary material for: Seasonal climate conditions impact the effectiveness of improving photosynthesis to increase soybean yield
Source: Field Crops Res. 2023 May 15;296:108907. doi: 10.1016/j.fcr.2023.108907 (PMC10155077; doi:10.1016/j.fcr.2023.108907)
Supplement: Supplementary file 1 — Supplementary material. [file mmc1.docx]

Supplementary Materials for

Seasonal climate conditions impact the effectiveness of improving photosynthesis to increase soybean yield

Yufeng He^a^, Megan L. Matthews^a,b*^

^a^Carl R. Woese Institute for Genomic Biology, University of Illinois at Urbana-Champaign, Illinois 61801, U.S.A.

^b^Department of Civil and Environmental Engineering, University of Illinois at Urbana-Champaign, Illinois 61801, U.S.A.

*Corresponding author: Megan L. Matthews. Email: mlmatth2@illinois.edu

## A brief description on the Soybean-BioCro model

Soybean-BioCro consists of five key processes: Multilayer canopy properties, canopy C3 photosynthesis and leaf microclimate, carbon allocation and senescence, crop development, and soil-water processes. The diagram below (Fig. S1) is a simplified schematic of some of the key processes in the model, including 1) Leaf-level photosynthesis using a coupled FvCB and Ball-Berry stomatal conductance iteration solver that finds the Ci and assimilation at an equilibrium state; 2) 10-layer canopy integration including both sunlit and shaded assimilations and the Penman-Monteith evapotranspiration model; and 3) Growth development based on a set of photothermal functions and biomass partitioning using independent logistical functions for each of the organs. Leaf temperature is passed to the FvCB model for each layer and all Rubisco-, RuBP- and TPU-limited photosynthesis rates are temperature-dependent (BERNACCHI et al., 2003; Yang et al., 2016).

For a complete description including all model equations, please refer to the supplementary information in (Matthews et al., 2022).


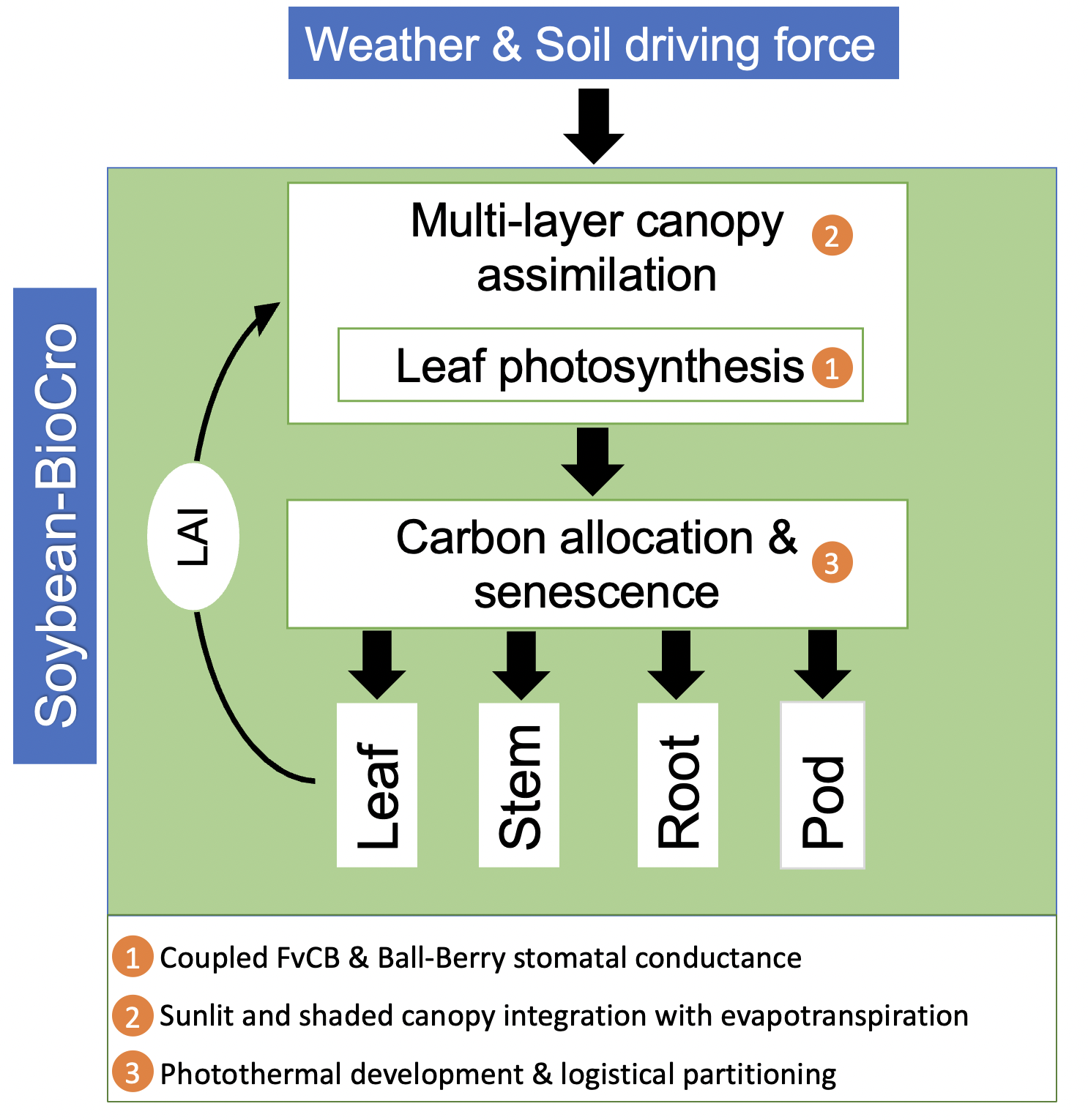


Figure S1 Main model processes in the Soybean-BioCro model.

## Other figures & tables


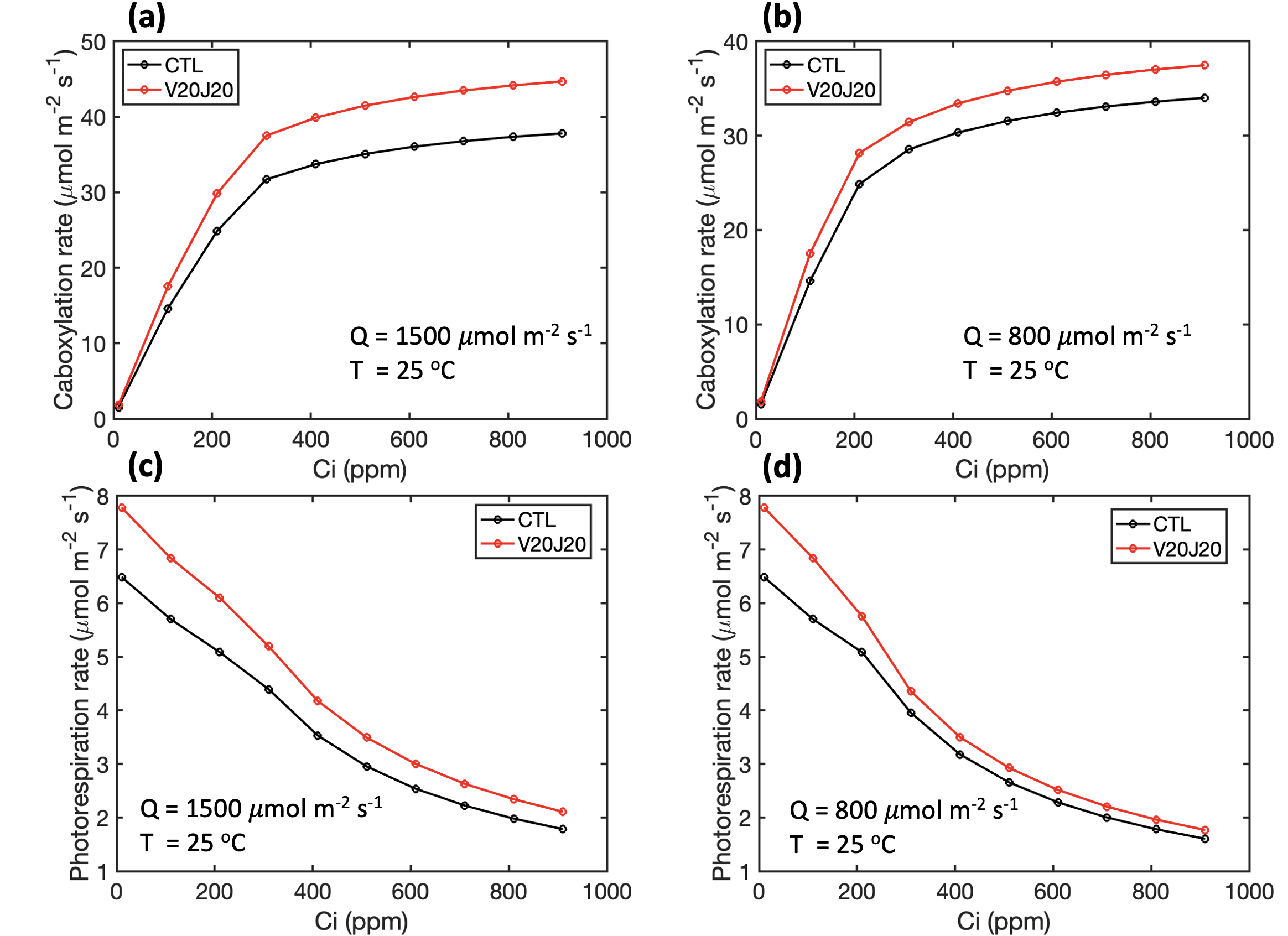


Figure S2 Modeled leaf-level carboxylation rate against Ci at two light conditions (a & b) for the control (CTL) and experiments with increased Vcmax and Jmax (V20J20). Similarly, modeled photorespiration rate against Ci (c & d). The model parameters used here are the same as the ones in Figure 5. The carboxylation rate shown here is calculated by excluding the photorespiration in the FvCB model.

Table 1 This table lists the numbers that are represented in Fig. S2. The last row shows the ratio of the changes in photorespiration and carboxylation, where Δ = V20J20 – CTL.


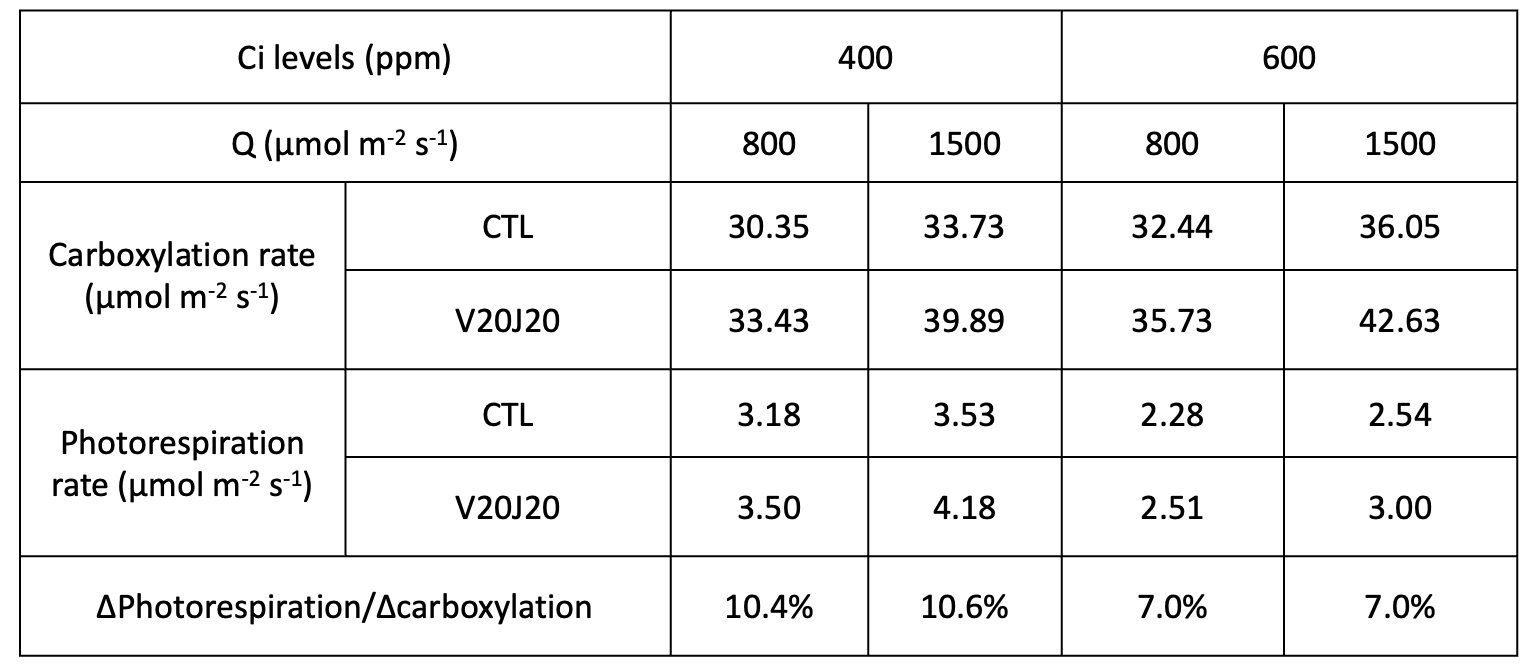


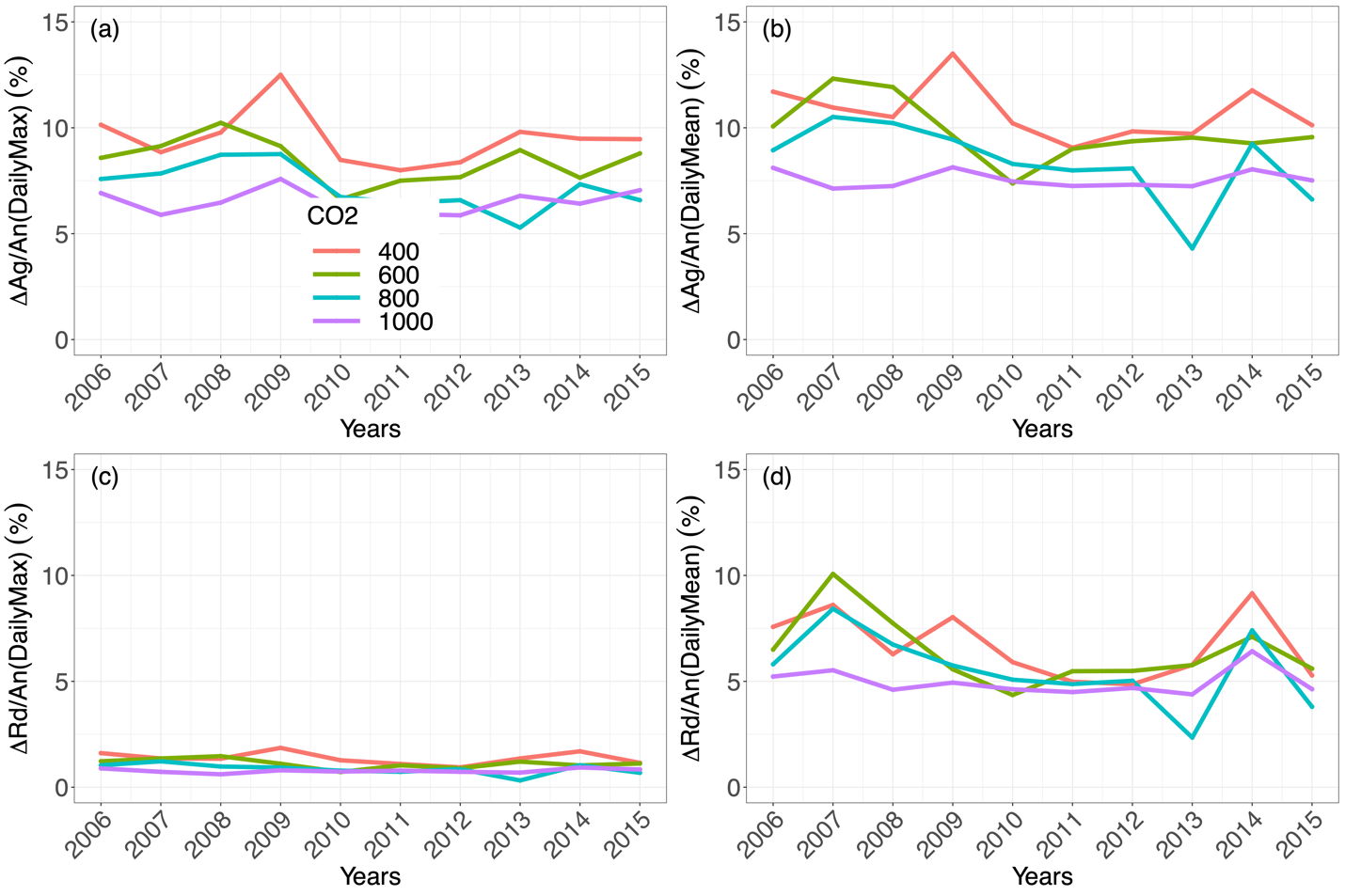


Figure S3 Contributions of the canopy-level gross assimilation (Ag) and dark respiration (Rd) to the changes in daily maximum and mean An as shown in Fig. 3. The percentages represent the proportion of either ΔAg or ΔRd over the control’s An, where Δ = V20J20 – CTL. (a) Seasonal average of ΔAg/An for daily maximum An; (b) Seasonal average of daily mean ΔAg/An; (c) Seasonal average of ΔRd/An for daily maximum An; (b) Seasonal average of daily mean ΔRd/An. Note that An = Ag – Rd and Rd thus contributes negatively to the An.


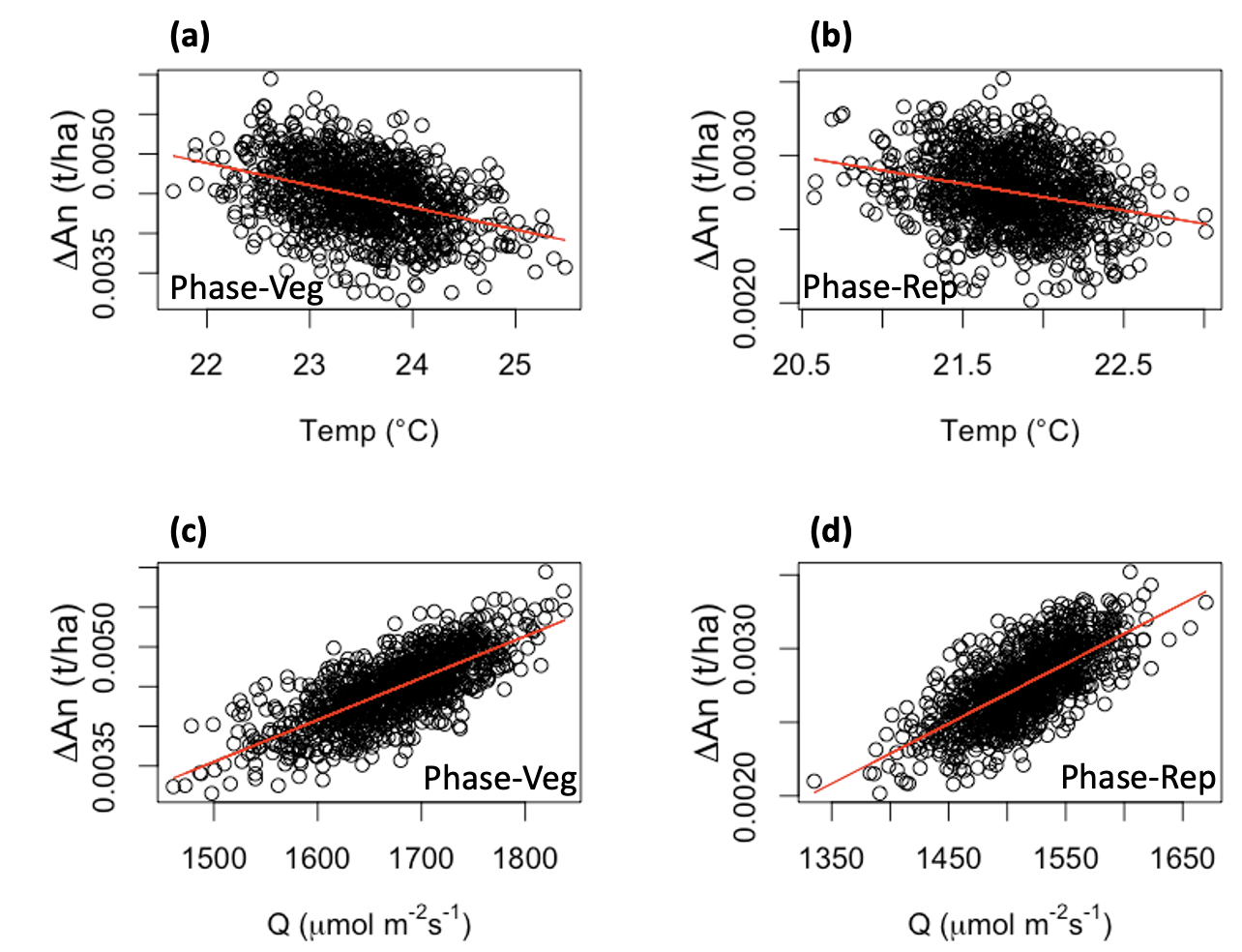


Figure S4 Relationships between the maximum ΔA_n_ and two climate variables, temperature (temp) and radiation (Q) for the 1000 bootstrap samples. The whole growing was separated into two growth periods, the vegetative and reproductive phases.


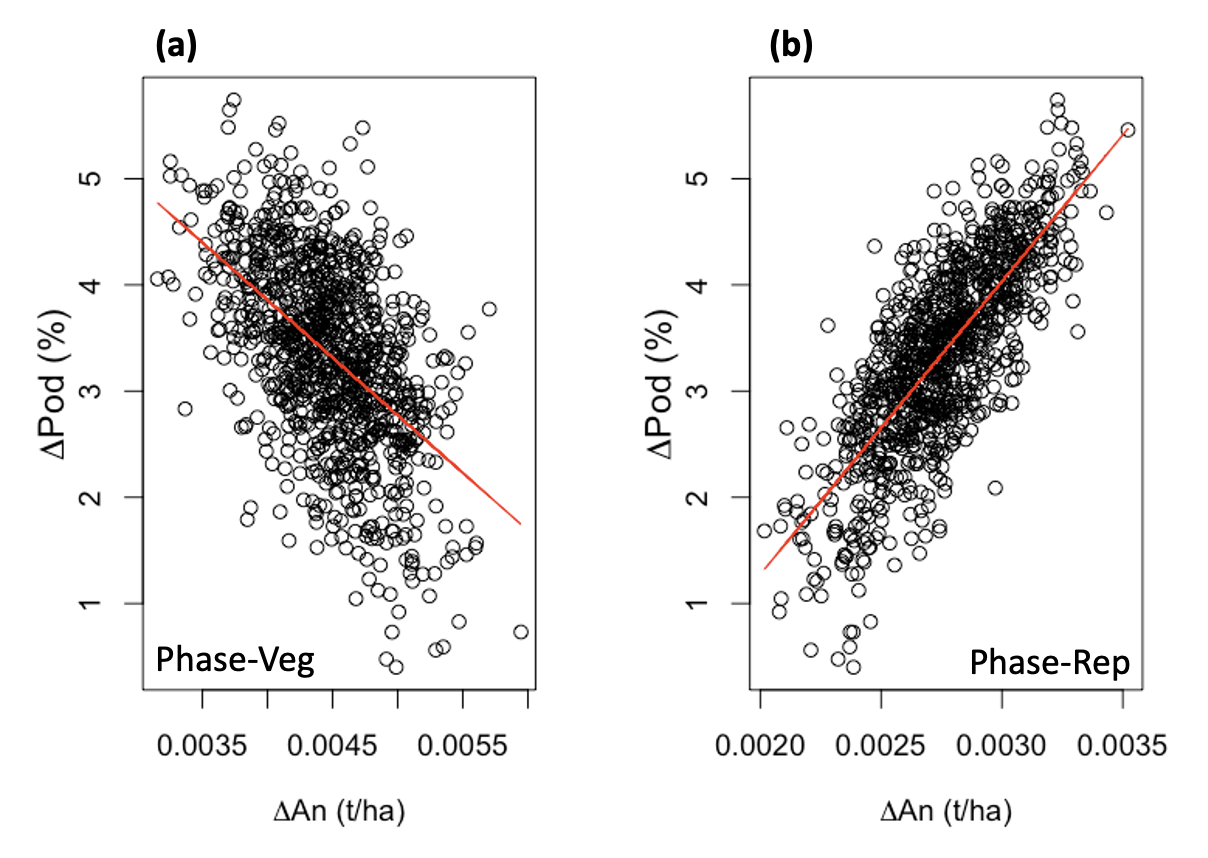


Figure S5 Relationships between the maximum ΔA_n_ and ΔPod for the 1000 bootstrap samples. The whole growing was separated into two growth periods, the vegetative and reproductive phases.

## References:

BERNACCHI, C.J., PIMENTEL, C., LONG, S.P., 2003. *In vivo* temperature response functions of parameters required to model RuBP-limited photosynthesis. Plant Cell Environ 26, 1419–1430. https://doi.org/10.1046/j.0016-8025.2003.01050.x

Matthews, M.L., Marshall-Colón, A., McGrath, J.M., Lochocki, E.B., Long, S.P., 2022. Soybean-BioCro: a semi-mechanistic model of soybean growth. In Silico Plants 4. https://doi.org/10.1093/insilicoplants/diab032

Yang, J.T., Preiser, A.L., Li, Z., Weise, S.E., Sharkey, T.D., 2016. Triose phosphate use limitation of photosynthesis: short-term and long-term effects. Planta 243, 687–698. https://doi.org/10.1007/s00425-015-2436-8
